# Patients’ perceptions of visual impairment associated with smoking: A cross-sectional study of a United Kingdom tertiary eye centre

**DOI:** 10.1177/11206721211020647

**Published:** 2021-05-31

**Authors:** Diya Baker, Onyinye Akpenyi, Haris Shahzad, Faye Mellington

**Affiliations:** 1 Institute of Continuing Education, University of Cambridge, Madingley Hall, Madingley, Cambridge, UK; 2 Birmingham Midland Eye Centre, Birmingham, UK; 3 College of Medical and Dental Sciences, University of Birmingham, Edgbaston, Birmingham, UK

**Keywords:** Preventive medicine/screening, socioeconomics and education in medicine/ophthalmology, lens/cataract, age-related macular degeneration, retina, thyroid eye disease, orbital disease, retinopathy of prematurity, diabetic retinopathy, uveitis

## Abstract

Smoking is a well-established risk factor for several eye disorders including cataracts and age-related macular degeneration. While many individuals are informed of the various adverse health effects, there is limited research into patients’ awareness of the relationship between smoking and eye disease and the potential impact this might have on reducing smoking behaviour. Our findings document the low level of awareness of the risk of blindness from smoking at a tertiary eye unit in the United Kingdom and highlight the need for increased involvement from eye care professionals, alongside health campaigns to educate the public of this consequence of smoking.

Smoking is a well-established risk factor for several eye disorders including cataracts, age-related macular degeneration, and retinal vascular occlusion. It affects the eye largely through ischaemic and oxidative mechanisms.^1^ Whilst the role of smoking in vascular injury in other organs is widely accepted, there is limited research into patient-specific awareness of the relationship between smoking and eye disease, and the potential impact of this knowledge on reducing smoking. Our aim was to document patient awareness of this association amongst patients attending Eye Casualty at The Birmingham and Midland Eye Centre (BMEC), one of the largest eye hospitals in Europe. We carried out a cross-sectional survey using a 21-question instrument given to patients over a 12-week period to establish smoking status, awareness of the connection between smoking and eye disorders, and delivery of smoking cessation intervention.

Of the 232 patients who completed the survey, almost half were smokers or ex-smokers. A third of patients either smoked, vaped or passive-smoked (18%, 6%, 12%), and 13% were ex-smokers. With regard to perceptions of smoking-related visual impairment, less than 1 in 10 respondents were aware that smoking was a cause of blindness. Around a quarter knew it was a risk factor for ocular diseases such as cataracts and diabetic retinopathy. Only 7% had been asked about their smoking status by a healthcare professional, some of whom had received smoking cessation interventions such as counselling or nicotine patches, mostly by General Practitioners.

Our findings (Fig. 1) indicate that regardless of smoking status, most patients are unaware that smoking may cause blindness and other ocular conditions. These results corroborate with previous studies including a 2011 international tobacco control project by Kennedy et al.,^2^ and a 2005 cross-sectional study by Bidwell et al.^3^ By far the largest eye casualty in the region, we believe our results from the BMEC are reflective of regional knowledge about smoking and eye health, and potentially similar to other large city-based populations in Europe, where there is a greater prevalence of smoking.^4^

**Figure 1.**
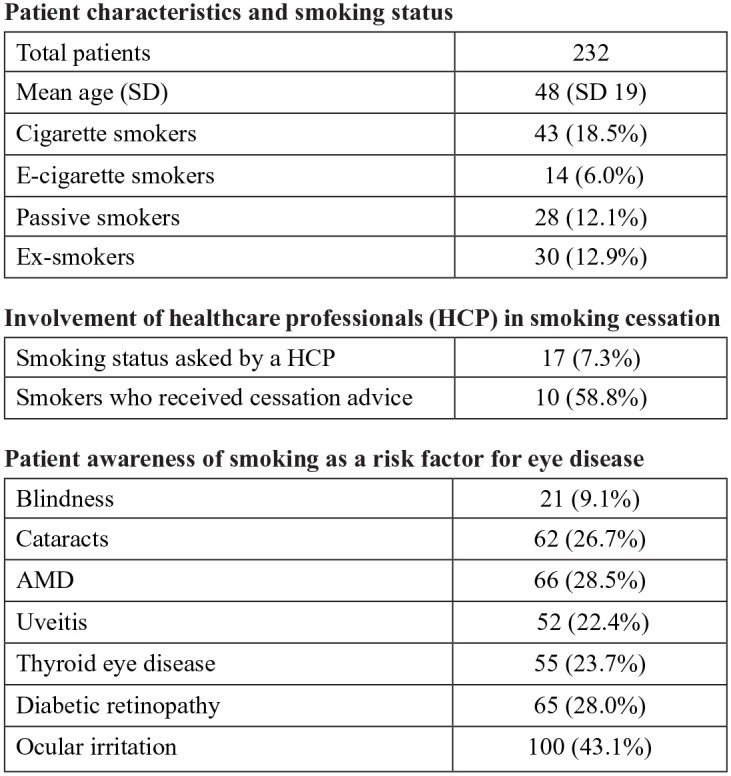
Table of characteristics, smoking status, receipt of cessation interventions, and awareness of smoking-related ocular disease and vision impairment.

The findings suggest insufficient publicly available information on the issue and missed opportunities by health professionals to educate patients about the dangers of smoking on eye health. Central and branch retinal artery occlusion in particular have been shown to correlate with a significantly higher prevalence of smoking and cardiovascular disease.^5^

Many cardiovascular, respiratory, and diabetes clinics incorporate formal smoking cessation advice into their service repertoire, and our findings highlight the need for eye clinic and optometry services to follow suit. The efficacy of patient education in this area is proven: visual impairment as a motivator to quit smoking and vaping should inform public health campaigns in order to engage the community in tobacco control. In Australia, for example, a study by Carroll and Rock^6^ described how a television campaign highlighting smoking and blindness was more effective than similar adverts linking it to heart disease and stroke. Other tools may include educational displays in Eye Casualty and outpatient waiting rooms, readily available information leaflets, and easily accessible smoking cessation support.

We advocate all such strategies to publicise the impact of smoking and vaping on eye health, alongside the incorporation of routine smoking cessation advice in eye consultations.
